# Hippocampal adenosine-to-inosine RNA editing in sepsis: dynamic changes and influencing factors

**DOI:** 10.1093/braincomms/fcae260

**Published:** 2024-08-08

**Authors:** Yun-Yun Jin, Ya-Ping Liang, Zhi-Yuan Wei, Wei-Jia Sui, Jian-Huan Chen

**Affiliations:** Laboratory of Genomic and Precision Medicine, Wuxi School of Medicine, Jiangnan University, Wuxi, Jiangsu 214122, China; Joint Primate Research Center for Chronic Diseases, Jiangnan University and Institute of Zoology, Guangdong Academy of Sciences, Wuxi School of Medicine, Jiangnan University, Wuxi, Jiangsu 214122, China; Jiangnan University Brain Institute, Wuxi School of Medicine, Jiangnan University, Wuxi, Jiangsu 214122, China; Laboratory of Genomic and Precision Medicine, Wuxi School of Medicine, Jiangnan University, Wuxi, Jiangsu 214122, China; Joint Primate Research Center for Chronic Diseases, Jiangnan University and Institute of Zoology, Guangdong Academy of Sciences, Wuxi School of Medicine, Jiangnan University, Wuxi, Jiangsu 214122, China; Jiangnan University Brain Institute, Wuxi School of Medicine, Jiangnan University, Wuxi, Jiangsu 214122, China; Laboratory of Genomic and Precision Medicine, Wuxi School of Medicine, Jiangnan University, Wuxi, Jiangsu 214122, China; Joint Primate Research Center for Chronic Diseases, Jiangnan University and Institute of Zoology, Guangdong Academy of Sciences, Wuxi School of Medicine, Jiangnan University, Wuxi, Jiangsu 214122, China; Jiangnan University Brain Institute, Wuxi School of Medicine, Jiangnan University, Wuxi, Jiangsu 214122, China; Laboratory of Genomic and Precision Medicine, Wuxi School of Medicine, Jiangnan University, Wuxi, Jiangsu 214122, China; Joint Primate Research Center for Chronic Diseases, Jiangnan University and Institute of Zoology, Guangdong Academy of Sciences, Wuxi School of Medicine, Jiangnan University, Wuxi, Jiangsu 214122, China; Jiangnan University Brain Institute, Wuxi School of Medicine, Jiangnan University, Wuxi, Jiangsu 214122, China; Laboratory of Genomic and Precision Medicine, Wuxi School of Medicine, Jiangnan University, Wuxi, Jiangsu 214122, China; Joint Primate Research Center for Chronic Diseases, Jiangnan University and Institute of Zoology, Guangdong Academy of Sciences, Wuxi School of Medicine, Jiangnan University, Wuxi, Jiangsu 214122, China; Jiangnan University Brain Institute, Wuxi School of Medicine, Jiangnan University, Wuxi, Jiangsu 214122, China

**Keywords:** RNA editing, sepsis-associated encephalopathy, hippocampus, sex, age

## Abstract

Sepsis-associated encephalopathy is a diffuse brain dysfunction secondary to infection. It has been established that factors such as age and sex can significantly contribute to the development of sepsis-associated encephalopathy. Our recent study implicated a possible link between adenosine-to-inosine RNA editing and sepsis-associated encephalopathy, yet the dynamics of adenosine-to-inosine RNA editing during sepsis-associated encephalopathy and how it could be influenced by factors such as age, sex and antidepressants remain uninvestigated. Our current study analysed and validated transcriptome-wide changes in adenosine-to-inosine RNA editing in the hippocampus of different septic mouse models. Seventy-four sites in 64 genes showed significant differential RNA editing over time in septic mice induced by caecal ligation and perforation. The differential RNA editing might contribute to the RNA expression regulation of the edited genes, with 42.2% differentially expressed. These differentially edited genes, especially those with missense editing, such as glutamate receptor, ionotropic, kainate 2 (*Grik2*, p.M620V), filamin A (*Flna*, p.S2331G) and capicua transcriptional repressor (*Cic*, p.E2270G), were mainly involved in abnormal social behaviour and neurodevelopmental and psychiatric disorders. Significant effects of age and sex were also observed on sepsis-associated RNA editing. Further comparison highlighted 40 common differential RNA editing sites that caecal ligation and perforation-induced and lipopolysaccharide-induced septic mouse models shared. Interestingly, these findings demonstrate temporal dynamics of adenosine-to-inosine RNA editing in the mouse hippocampus during sepsis, add to the understanding of age and sex differences in the disease and underscore the role of the epigenetic process in sepsis-associated encephalopathy.

## Teaser

Jin *et al*. report altered hippocampal adenosine-to-inosine RNA editing in different sepsis mouse models. Age influences temporal hippocampal adenosine-to-inosine RNA editing changes during sepsis, with more evident effects than sex. Additionally, fullerenol pre-treatment partially rescued sepsis-associated RNA editing alterations.

## Graphical Abstract

**Graphical Abstract:**
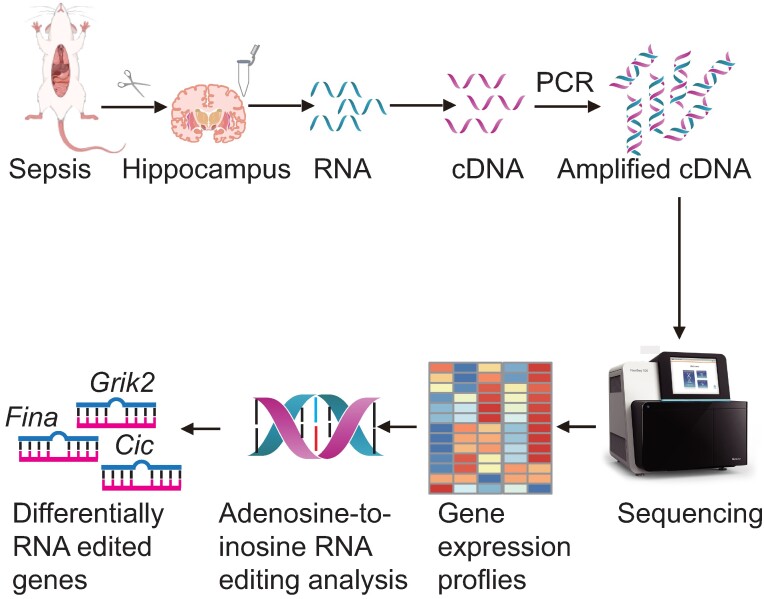

## Introduction

Sepsis-associated encephalopathy (SAE) is the most common brain dysfunction in sepsis.^1^ Up to 70% of patients affected with sepsis could develop SAE, which may cause poor outcomes. SAE could be associated with increased mortality and long-term cognitive impairment.^2^ The hippocampus is the most important and most investigated brain region during sepsis and is the primary brain region responsible for memory and emotional control.^3,4^ Studies have shown that sepsis can lead to cognitive impairment and memory deficits, which are closely linked to hippocampal dysfunction.^5-8^ Sex differences and effects of age have been observed in sepsis and SAE.^9^ A consistently higher risk of sepsis and SAE was reported in men.^10^ Sepsis is more frequent among elderly adults, and its incidence increases with age, especially over 65. Elderly sepsis patients could also have higher severity and mortality.^11^

Emerging evidence from microbiology and immunology studies suggests a substantial role of epigenetic regulation in the pathogenesis of sepsis.^12^ Epigenetics may be crucial in essential aspects of sepsis, such as acute inflammation, immune suppression and host–pathogen interaction.^13^ As one of the critical epigenetic processes, RNA editing, especially adenosine-to-inosine (A-to-I) RNA editing, plays an essential regulatory role in inflammatory diseases and neurological diseases.^14^ The RNA editing enzyme ADAR, highly expressed in the small intestine of septic mice, has been reported to play a protective role against sepsis,^15^ providing a new therapeutic target for sepsis.^16^ However, there have been limited studies on A-to-I RNA editing in sepsis and SAE. Our recent study implicated altered A-to-I RNA editing in the post-mortem cortex tissues of SAE compared with controls. Nevertheless, the dynamics of A-to-I RNA editing during SAE and the influence of sex and age on it remain unclear.

Our current study analysed the association of A-to-I RNA editing in the hippocampus with sepsis in different sepsis mouse models and evaluated the possible effects of sex and age on this process. In addition, fullerenol, a water-soluble derivative of fullerene with antidepressant-like properties,^17^ was examined for its impact on sepsis-associated RNA editing in an lipopolysaccharide (LPS)-induced depressive mouse model.

## Materials and methods

### Ethics statement

This article does not require any ethical approval.

### Hippocampus RNA-seq data sets of septic mouse models

Hippocampus RNA sequencing FASTQ files of three data sets (BioProject accession numbers PRJNA529377, PRJNA827615 and PRJNA894332) were retrieved from NCBI’s Sequence Read Archive (SR) database. The data set PRJNA529377 contained 48 C57BL/6 mice of controls and septic mice on Day 1 (D1) and Day 4 (D4) after caecal ligation and perforation (CLP) treatment (*N* = 16 each for the control, D1 and D4 groups, respectively).^18^ Each group contained four subgroups of young (3–5 months) and old (18–22 months) male and female mice (*N* = 4 for each subgroup). The sepsis induction by CLP in mice was described in the original study.^18^ In brief, a midline laparotomy was performed, the caecum was ligated ∼1 cm from its tip, and caecal puncture was conducted using a 22-gauge needle. The data set PRJNA827615 contained twelve 8–10-week-old male C57BL/6J mice, including six at D1 after peritoneal injection of LPSs and six controls.^4^ The data set PRJNA894332 contained nine 8-week-old male C57BL/6J mice, including three controls, three with peritoneal injection of LPS and three pre-treated with fullerenol and peritoneal injection of LPS.^17^ The fullerenol treatment regimen was described in the original study. In brief, control mice were intraperitoneally injected with normal saline for 7 days. The other mice received 2 days of fullerenol (10 mg/kg) or saline treatment, followed by LPS (1 mg/kg) or saline injections for 5 days.

### RNA-seq data alignment

The FASTQ files were then processed as described in our previous studies.^19^ After the adaptor and low-quality sequences were removed using FASTP, the processed reads were mapped to the mouse reference genome sequence (UCSC mm10) using RNA STAR (version 2.7.0e).^20^ Uniquely mapped reads were obtained using SAMtools (version 1.9),^21^ and base quality scores were recalibrated using GATK (version 4.1.3).^22^

### Identification and annotation of RNA editing

A pipeline based on VarScan (version 2.4.4)^23^ and the Ensembl Variant Effect Predictor (VEP),^24^ which was described previously, was used to identify and annotate RNA editing sites.^19^ In addition, only A-to-G single nucleotide variations (SNVs) annotated as known editing variants in the REDIportal V2.0 database^25^ or with an averaged editing level ≥5% in at least one subgroup and observed in at least two samples were retained as high-confidence variants.

### Normalized gene expression

The Rsubread package of the R language was used to calculate pseudo-counts of the RNA expression^26^ and normalized gene expression levels (transcript per million).

### Protein–protein interaction network construction and gene function enrichment analysis

Protein–protein interaction (PPI) network was constructed using the STRING database website. Gene function enrichment analysis was performed using DAVID,^27^ and Monarch was implemented on the STRING database website.

### Functional enrichment analysis

Gene Ontology and Kyoko Encyclopedia of Genes and Genomes (KEGG) pathway analyses were conducted using online prediction tools, including DAVID (https://david.ncifcrf.gov/tools.jsp), Enrichr (https://maayanlab.cloud/Enrichr/) and an online tool (http://www.bioinformatics.com.cn/).^27^ Significance was determined based on a false discovery rate (FDR) <0.05.

### RNA-binding protein binding site prediction

To further understand the potential functional implications of RNA editing, RBPmap (http://rbpmap.technion.ac.il) was used to predict RNA-binding protein (RBP) sites that coincided with RNA editing sites.^28^

### Statistical analysis

The generalized linear model (GLM) and likelihood ratio test (LRT) were used to compare the intergroup levels of RNA editing or gene expression and calculate the empirical *P*-values (*P*_GLM_). For empirical *P*_GLM_ < 0.05, an additional Fisher’s exact test was conducted to calculate *P*-values (*P*_Fisher_) by comparing the total counts of the reference and alternative alleles among groups. The Benjamini–Hochberg method for FDR adjusted empirical *P*-values for multiple comparisons. Spearman correlation was used to analyse the cis-regulatory effects of RNA editing on the edited gene expression and to calculate the correlation coefficients (*r*) and *P*-values.

## Results

### RNA editing identified from mouse hippocampal transcriptome

**Figure 1:**
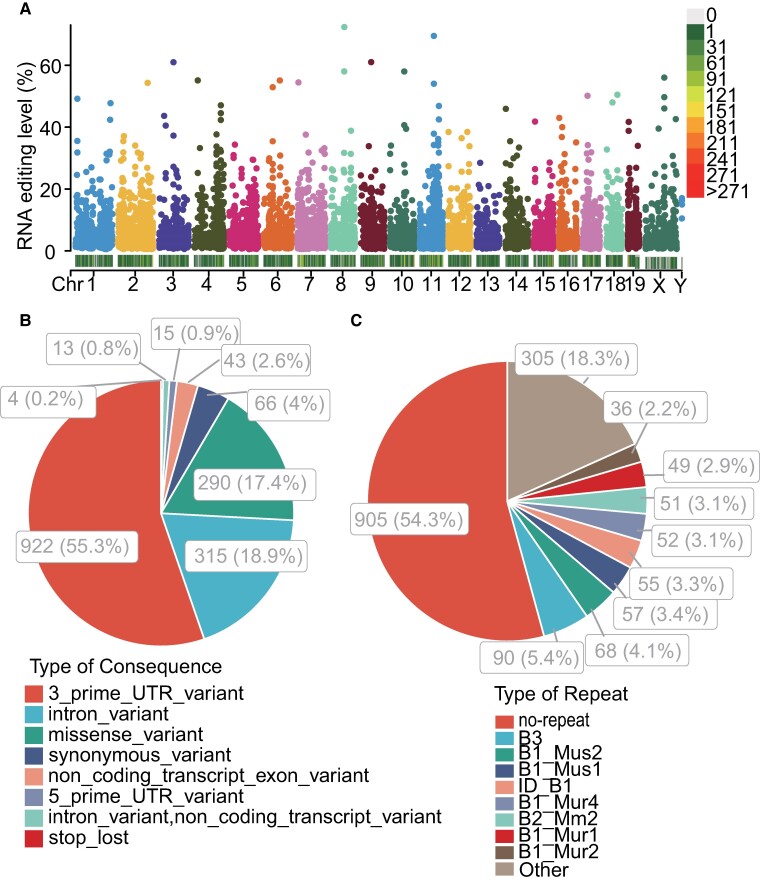
**Epitranscriptomic analysis of hippocampal A-to-I RNA editing in septic and control mice in the current study.** (**A**) Manhattan and density plot showing the distribution of the A-to-I RNA editing sites in the hippocampus of D1 and D4 sepsis groups and the control group (*N* = 16, respectively). Statistics of A-to-I RNA editing variants’ functional consequences (**B**) and overlapping repeats (**C**). Repeat types in the murine genome contain the SINE families of B1, B2, B3 and ID elements. Chr, chromosome.

From the transcriptomic data of all samples in the mouse hippocampus data set PRJNA529377, a total of 1668 high-confidence A-to-I RNA editing sites were found in 983 genes (Fig. 1A), including 922 (55.3%) 3′ untranslated region (3′UTR), 315 (18.9%) intronic, 290 (17.4%) missense, 66 (4%) synonymous, 43 (2.6%) non-coding transcript exonic, 15 (0.9%) 5′UTR, 13 (0.8%) non-coding transcript intronic and 4 (0.2%) stop–loss variants (Fig. 1B, Supplementary Table 1). The repeat most overlapped with these RNA editing sites was the B1 family (Fig. 1C).

### Temporal dynamics of hippocampal RNA editing during sepsis

**Figure 2:**
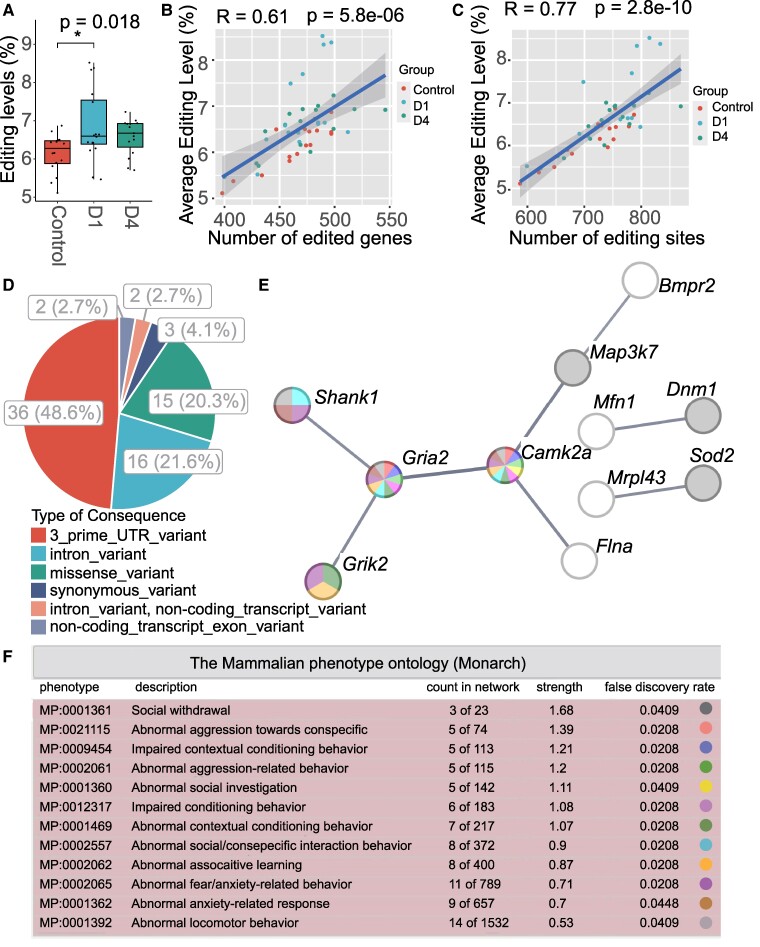
**Temporal dynamics of sepsis-associated A-to-I RNA editing in the mouse hippocampus.** (**A**) The sample-wide average level of RNA editing among the control, D1 and D4 sepsis groups (*N* = 16, respectively). *P < 0.05. Overall and *post hoc P*-values are calculated using the ANOVA and Tukey tests. (**B**, **C**) The Pearson correlation analysis of RNA editing level with both the numbers of edited genes (**B**) and editing sites (**C**) in the hippocampal samples. (**D**) The functional categories of DRE sites. (**E**) PPI network of genes differentially edited in the hippocampus during sepsis. The network is constructed based on the STRING database. (**F**) Mammalian phenotypes enriched by the differentially edited genes. The enrichment analysis is conducted according to the Monarch database.

We then analysed the temporal changes of A-to-I RNA editing in the hippocampus by comparing the control, D1 and D4 groups using the ANOVA method. The transcriptome-wide average level of A-to-I RNA editing showed a significant difference among the three groups, which was higher in the two septic groups than in the controls (Fig. 2A, Supplementary Table 1).

To identify differential RNA editing (DRE) in sepsis, individual sites with an editing level of ≥5% editing in at least one subgroup (young male, young female, old male and old female) of controls, D1 or D4 were included in the comparison among different groups. Our study showed a significant difference in the average level of A-to-I RNA editing among groups, especially between the control and D1 septic groups (Fig. 2A). This suggests that sepsis could induce global changes in the hippocampal RNA editing landscape.

In our study, the number of RNA editing sites and edited genes was also higher in the septic groups than in controls, although it did not reach statistical significance (data not shown). The correlation between the average level of editing and the number of edited genes or editing sites is an interesting point. We performed Pearson correlation analysis and found a significant correlation of the average RNA editing level with both the numbers of edited genes (*r* = 0.61, *P* = 5.8e^−06^) and editing sites (*r* = 0.77, *P* = 2.8e^−10^) in the hippocampal samples (Fig. 2B and C).

**Figure 3:**
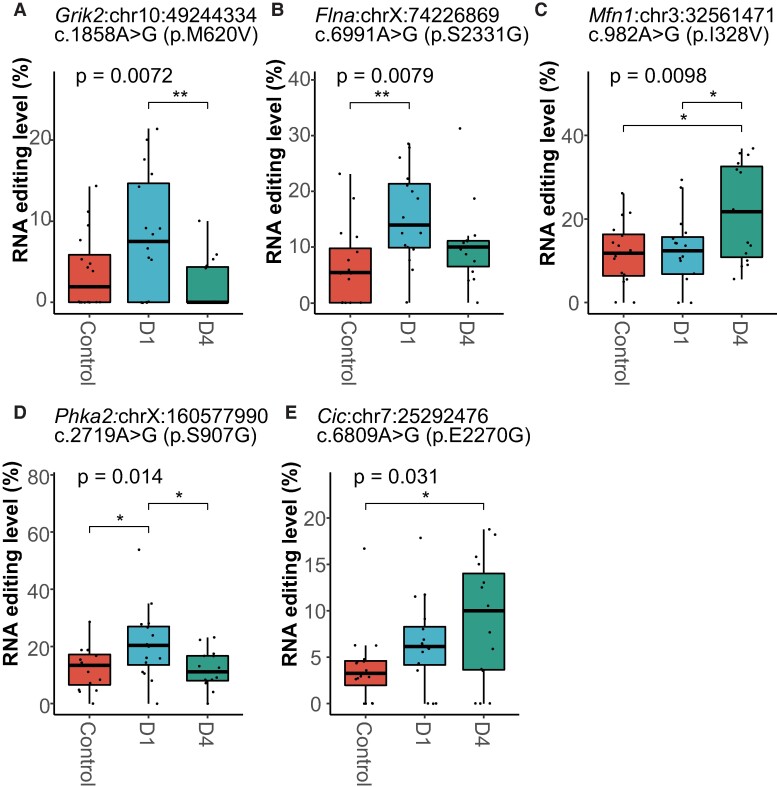
**Missense sites showed DRE in the mouse hippocampus during CLP-induced sepsis.** (**A–E**) *Grik2* (p.M620V), *Flna* (p.S2331G), *Mfn1* (p.I328V), *Phka2* (p.S907G) and capicua transcriptional repressor (*Cic*, p.E2270G) A-to-I RNA editing level, respectively (*N* = 16, respectively). *P < 0.05; **P < 0.01. *P*-values are calculated using the GLM model and LRT.

Our results identified 74 DRE sites in 64 genes (Supplementary Table 1), more than half of which could be divided into three clusters with different trends of editing changes (Supplementary Fig. 1). The largest contained sites first upregulated at D1 and then downregulated at D4. The moderate cluster had sites upregulated significantly at D4. The smallest cluster contained sites downregulated at D1 and D4. The repeat most overlapped with these DRE sites was the B1 family (Supplementary Fig. 2). Most of these DRE sites were located within 3′UTR or resulted in a missense change (Fig. 2D). Notably, among the 15 missense DRE sites, 5 of them were found to show at least 5% intergroup difference in the editing level, including glutamate receptor, ionotropic, kainate 2 (*Grik2*, p.M620V), filamin A (*Flna*, p.S2331G), mitofusin 1 (*Mfn1*, p.I328V), phosphorylase kinase alpha 2 (*Phka2*, p.S907G) and capicua transcriptional repressor (*Cic*, p.E2270G) (Fig. 3, Supplementary Table 2).

In addition to protein-coding genes, three long non-coding RNA genes were also differentially edited, including myocardial infarction-associated transcript (*Miat*), small nucleolar RNA host gene 11 (*Snhg11*) and maternally expressed gene 3 (*Meg3*) (Supplementary Table 1).

The results found that 26 shared genes were differentially edited and differentially expressed (Supplementary Fig. 3). Correlation analysis was further performed to investigate the cis-regulatory effects of DRE on gene expression. Twelve DRE sites showed a significant correlation between the RNA editing level and the gene expression level (*P* < 0.05) (Supplementary Table 3).

To further understand the functional relevance of the temporal dynamics of the DRE, PPI network construction and mammalian phenotype enrichment analysis were performed using the STRING database with all differentially edited genes (Fig. 2E and F). Our results identified a cluster composed of seven genes, including glutamate ionotropic receptor AMPA type subunit 2 (*Gria2*), calcium/calmodulin-dependent protein kinase type II subunit alpha (*Camk2a*), SH3 and multiple ankyrin repeat domains protein 1 (*Shank1*), *Grik2*, *Flna*, mitogen-activated protein kinase kinase kinase 7 (*Map3k7*) and bone morphogenetic protein receptor type-2 (*Bmpr2*) (Fig. 2E). Monarch results showed that these differentially edited genes were mainly enriched in abnormal behaviours related to social withdrawal, aggression, investigation, learning and fear/anxiety (Fig. 2F). Twenty differentially edited genes were related to at least one of these phenotypes (Supplementary Table 4). Notably, the seven-gene cluster was also involved in most abnormal phenotypes.

**Figure 4:**
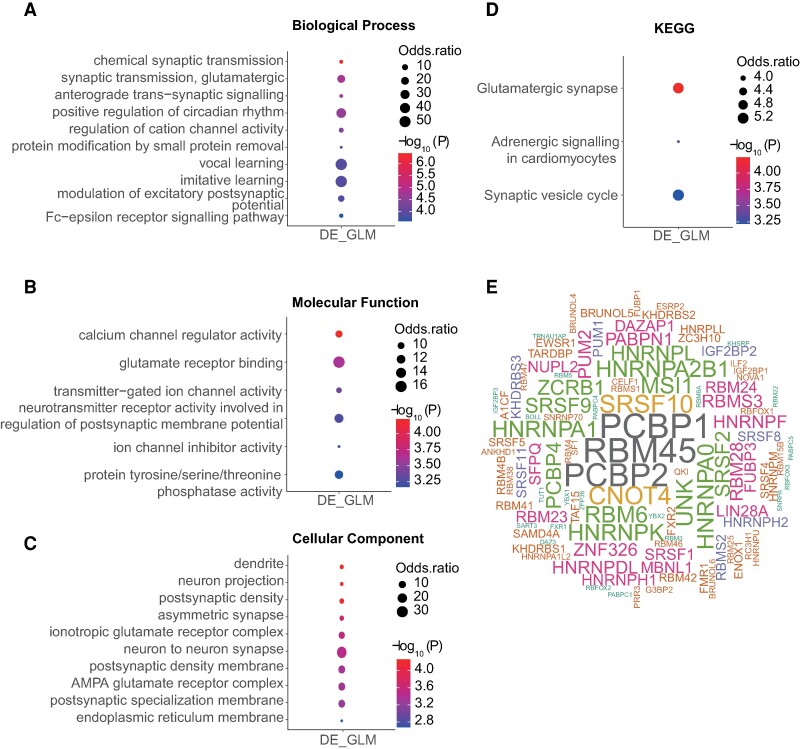
**Functional relevance of DRE A-to-I RNA editing in the mouse hippocampus.** (**A–D**) The enrichment analysis results of biological processes (**A**), molecular functions (**B**), cellular components (**C**) and KEGG pathway (**D**) enriched by differentially edited genes are shown. (**E**) Wordcloud plot of RBPs with binding sites overlapped with SAE-associated DRE. GLM, generalized linear model; LRT, likelihood ratio test.

Gene Ontology and KEGG pathway functional enrichment analysis of the DRE genes was then used to understand the impact of A-to-I RNA editing changes on biological functions. The DRE genes were mainly involved in biological processes related to chemical synaptic transmission, synaptic transmission, glutamatergic and anterograde trans-synaptic signalling (Fig. 4A); molecular functions mainly related to calcium channel regulator activity, glutamate receptor binding and transmitter-gated ion channel activity (Fig. 4B); cellular components including the dendrite, neuron projection, postsynaptic density and asymmetric synapse (Fig. 4C); and KEGG pathways mainly related to glutamatergic synapse (Fig. 4D).

The RBPmap website was then used to predict RBP binding sites that overlapped with DRE sites. The top three RBPs ranked by their binding frequency to the DRE sites were RNA-binding protein 45 (RBM45), poly(RC) binding protein 1 (PCBP1) and PCBP2 (Fig. 4E).

### Effects of age and sex on hippocampal RNA editing changes during sepsis over time

**Figure 5:**
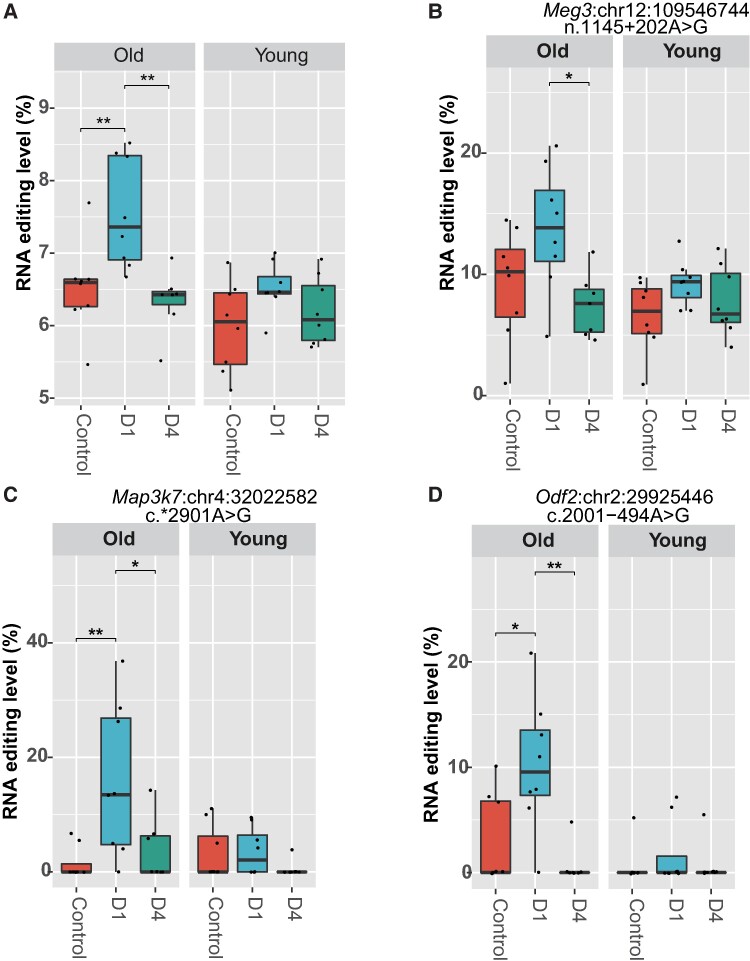
**The age effect on sepsis-associated A-to-I RNA editing in the mouse hippocampus.** (**A**) The age effect on the average level of A-to-I RNA editing among groups. The age-dependent RNA editing of *Meg3*:chr12:109546744 (**B**), *Map3k7*:chr4:32022582 (**C**) and *Odf2*:chr2:29925446 (**D**) are shown. The overall *P*-value is calculated using the GLM model and LRT with age (young or old, each containing three subgroups of control, D1 and D4, *N* = 8 for each subgroup) as a covariate. *P < 0.05; **P < 0.01. The *post hoc* analysis is conducted using the Tukey test. GLM, generalized linear model; LRT, likelihood ratio test.

Our data indicate that A-to-I RNA editing undergoes dynamic changes during sepsis. We then examined how age and sex may influence it. A significant effect of age on the transcriptome-wide average level of A-to-I RNA editing during sepsis was observed (*P* = 0.0015, Fig. 5A). Compared to young mice, a more intense upregulation of average A-to-I RNA editing level was found in old mice. In contrast, no such effect of sex was found on the transcriptome-wide average level of A-to-I RNA editing (*P* > 0.05, data not shown). Further analysis was performed to evaluate the effects of age and sex on individual DRE sites using GLM with age and sex as covariates. Our results showed that 14 DRE sites could be influenced by either age or sex (Table 1). Notably, an editing site in *Map3k7* and another editing site in *Meg3* were influenced by both factors (Table 1). Among them, the RNA editing levels of *Meg3*:chr12:109546744 (Fig. 5B), *Map3k7*:chr4:32022582 (Fig. 5C) and *Odf2*:chr2:29925446 (Fig. 5D) showed significant age-dependent change patterns similar to the average RNA editing.

**Table 1:** Effects of age and sex on the DRE in the hippocampus during sepsis

No.	Site ID	Gene	Gene biotype	Description	Consequence	cDNA change	Protein change	*P*_sex_	*P*_age_
1	Bmpr2:chr1:59874208	*Bmpr2*	Protein coding	Bone morphogenetic protein receptor, type II (serine/threonine kinase)	3′UTR variant	c.3723A>G		**0**.**0075**	0.8527
2	Odf2:chr2:29925446	*Odf2*	Protein coding	Outer dense fibre of sperm tails 2	Intron variant	c.2001-494A>G		0.1255	**0**.**0012**
3	Dnm1:chr2:32330638	*Dnm1*	Protein coding	Dynamin 1	Intron variant	c.1336-2591A>G		**0**.**0116**	**0**.**0462**
4	Dnm1:chr2:32330647	*Dnm1*	Protein coding	Dynamin 1	Intron variant	c.1336-2600A>G		**0**.**0284**	0.0955
5	Map3k7:chr4:32022582	*Map3k7*	Protein coding	Mitogen-activated protein kinase kinase kinase 7	3′UTR variant	c.2901A>G		**0**.**0131**	**0**.**0123**
6	Gm29609:chr5:31157715	*Gm29609*	Protein coding	Predicted gene 29609	Intron variant	c.2505+428A>G		**0**.**0169**	0.3277
7	Crtc1:chr8:70400641	*Crtc1*	Protein coding	CREB regulated transcription coactivator 1	Missense variant	c.602A>G	p.K201R	0.2615	**0**.**0034**
8	Timm29:chr9:21594388	*Timm29*	Protein coding	Translocase of inner mitochondrial membrane 29	3′UTR variant	c.550A>G		**0**.**0059**	0.5235
9	Zbtb4:chr11:69777687	*Zbtb4*	Protein coding	Zinc finger and BTB domain containing 4	Missense variant	c.1235A>G	p.E412G	**0**.**0043**	0.4765
10	Meg3:chr12:109546744	*Meg3*	lincRNA	Maternally expressed 3	Intron variant	n.1145+202A>G		**0**.**0046**	**0**.**0130**
11	Meg3:chr12:109547961	*Meg3*	lincRNA	Maternally expressed 3	Intron variant	n.1145+1419A>G		0.4046	**0**.**0163**
12	Pnck:chrX:73658122	*Pnck*	Protein coding	Pregnancy upregulated non-ubiquitously expressed CaM kinase	Missense variant	c.311A>G	p.E104G	0.2384	**0**.**0045**
13	Flna:chrX:74226869	*Flna*	Protein coding	Filamin, alpha	Missense variant	c.6991A>G	p.S2331G	**0**.**0467**	**0**.**0002**
14	Phka2:chrX:160577990	*Phka2*	Protein coding	Phosphorylase kinase alpha 2	Missense variant	c.2719A>G	p.S907G	**0**.**0222**	0.7046

Bold means a *P* < 0.05.

### Comparison of hippocampal DRE among CLP- and LPS-induced sepsis mouse models

**Figure 6:**
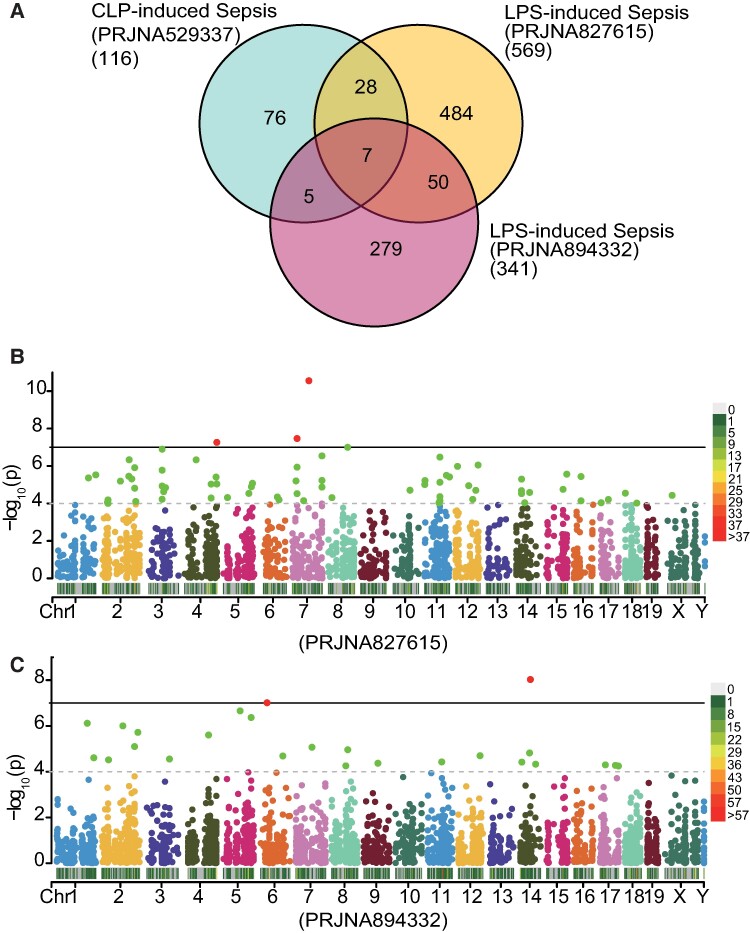
**Comparison of DRE among different data sets of septic mouse models induced by CLP and LPS.** (**A**) Venn plot showing DRE sites shared by different mouse model data sets. (**B**, **C**) Manhattan and density plots showing the distribution of the DRE sites in the hippocampus of two LPS-induced mice models (**B** for data set PRJNA827615 and **C** for data set PRJNA894332). The vertical straight line indicates a *P*-value of 10^−7^, and points above the line are with *P* < 10^−7^.

To further evaluate DRE in sepsis, we applied the same pipeline to identify DRE sites in the hippocampus between control and LPS-induced septic mice from two independent data sets (PRJNA827615 and PRJNA894332) and compare them with those observed in the CLP-induced septic mouse data set (PRJNA529377). The Venn plot in Fig. 6 compares DRE candidates with empirical *P*_GLM_ < 0.05. FDR correction was not applied to avoid over-correction when comparing multiple independent data sets. Forty DRE sites were differentially edited in the CLP-induced sepsis model and at least one of the two LPS-induced sepsis models. Notably, seven DRE sites were shared by all three data sets, namely *Nf2*:chr11:4767141, *Elfn2*:chr15:78685924, *Map3k7*:chr4:32022582, *Igf2*:chr7:142653273, *Pafah1b2*:chr9:45967489, *Ptprn*:chr1:75252513 and *Pafah1b2*:chr9:45967463 (Fig. 6A). In addition, another 50 DRE sites were shared by the two LPS data sets but not the CLP data set (Fig. 6A). Manhattan plots showed the distribution of DRE sites across the genome in the two LPS-induced sepsis mouse models (Fig. 6B and C). The results underlined that such common sepsis-induced RNA editing alterations in both types of sepsis models have an important role in the molecular pathogenesis of the disease.

### Fullerenol pre-treatment partially rescued sepsis-associated RNA editing alterations

**Figure 7:**
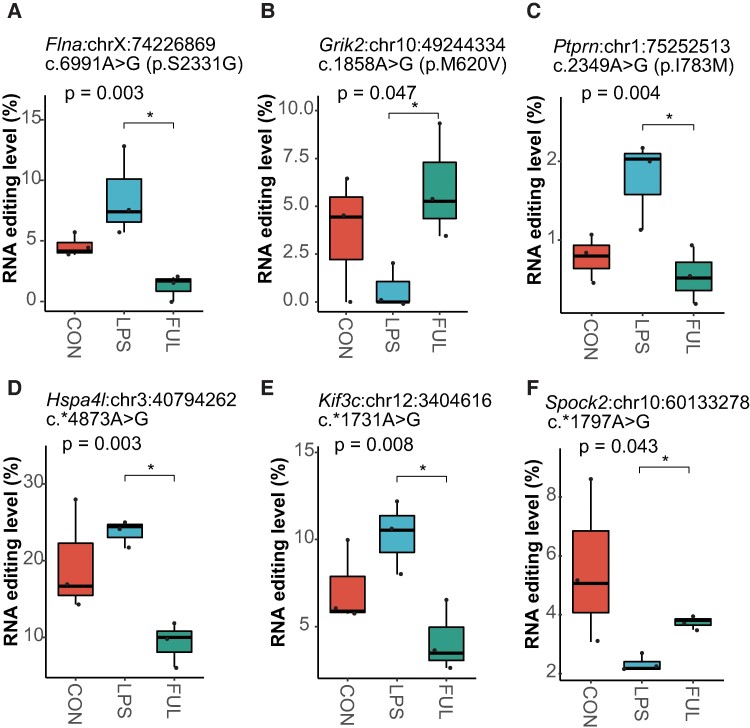
**Six sites with sepsis-associated RNA editing alterations are rescued by fullerenol pre-treatment in the hippocampus.** There are three missense editing including (**A**) *Flna* p.S2331G, (**B**) *Grik2* p.M620V and (**C**) *Ptprn* p.I783M and three 3′UTR editing including (**D**) *Hspa4l* c.*4873A>G, (**E**) *Kif3c* c.*1731A>G and (**F**) *Spock2* c.*1797A>G were rescued by fullerenol pre-treatment. Adult mice contained three groups, including CON, LPS and FUL (*N* = 3, each). *P < 0.05. *P*-values are calculated using the GLM model and LRT. CON, controls; LPS, mice receiving a peritoneal injection of LPS; FUL, mice pre-treated with fullerenol and receiving a peritoneal injection of LPS.

Recently, fullerenol was shown to have antidepressant-like characteristics in an LPS-induced depressive mouse model,^17^ in which fullerenol pre-treatment prevented a reduction in sucrose preference and downregulate neuroinflammation. We thus further examine the possible effect of fullerenol pre-treatment on hippocampal RNA editing in the same mouse model. Interestingly, six sites of sepsis-associated RNA editing alterations induced by LPS (Fig. 7A–F), especially the missense editing including *Grik2* p.M620V, *Flna* p.S2331G and *Ptprn* p.I783M, were rescued by fullerenol pre-treatment (Fig. 7A–C), which further underlined the relationship between RNA editing and SAE, especially depression.

## Discussion

Our recent study on septic patients’ brain tissues suggested that RNA editing could be involved in sepsis and SAE. In the current study, we further showed temporal dynamics of A-to-I RNA editing in the hippocampus in a sepsis mouse model, with dramatic effects of age and sex.

Our findings showed an elevated average A-to-I RNA editing level at D1 and D4 in the hippocampus in the CLP sepsis mouse model, suggesting the active involvement of RNA editing at the acute phase of sepsis. Our results also revealed dynamic yet divergent DRE changes, with at least three different clusters of DRE sites. Such findings thus pointed to a complex epigenetic regulation during sepsis. The biological impact of such A-to-I RNA editing changes remains unclear. RNA editing has been reported to impact the RNA abundance of genes related to the immune response^29^ and contributes to the proteomic diversity in cancers.^30^ Similarly, most sepsis-associated DRE SNVs identified in the hippocampus in the current study were 3′UTR and missense variants. A subset of these DRE SNVs might contribute to the expression regulation of the edited RNA, as 42.2% of the differentially edited genes exhibited differential expression during sepsis. In addition, a subset of these DRE sites showed potentially significant cis-regulatory effects on the expression of the edited genes. RNA editing has been reported to affect mRNA abundance and thus modulate immune pathways in cancers.^29^ RNA editing in the 3′UTR might regulate the RNA secondary structure stability or miRNA-binding efficiency of the edited genes.^31^ Another subset of these DRE SNVs was missense variants, which could impact the encoded proteins’ structure or function.

It was noted that several genes with DRE were previously reported or implicated to play a role in sepsis or SAE, such as superoxide dismutase 2 (*SOD2*), *Miat*, peptidylarginine deiminase 2 (*Padi2*), *Shank1*, transcription factor 4 (*Tcf4*) and kinesin family member 3 (*Kif3c*) (Supplementary Table 1). SOD2 is a mitochondrial enzyme that catalyzes the conversion of superoxide radicals to hydrogen peroxide and oxygen and thereby plays a crucial protective role against oxidative damage and inflammation in sepsis.^32^ SOD2 regulates lung oxidative damage and coagulation during sepsis, and enhancing its activity or delivery could be a potential therapeutic strategy.^32,33^*Padi2* encodes an enzyme that converts arginine residues to citrulline residues in proteins and plays a potential role in developing sepsis by modulating the inflammatory response. *Padi2* expression was significantly increased in septic patients and mice.^34,35^ Inhibition of PADI2 modulated macrophage polarization to the M2 phenotype^36^ and significantly improved survival in LPS-induced sepsis in mice.^37^ In addition, PADI2 contributes to oligodendrocyte differentiation and myelination and could thus be involved in motor and cognitive functions.^38^ The current study shows that *PADI2* was differentially expressed in the hippocampus during sepsis. These findings indicated that A-to-I RNA editing might play a role in sepsis and SAE by contributing to regulating genes involved in inflammation.

A-to-I RNA editing is associated with nervous system development and could be involved in neurological and psychiatric diseases such as developmental epileptic encephalopathy, depression and schizophrenia.^39-41^ In the current study, most of the differentially edited genes showed high-level expression in the brain. Moreover, these genes dynamically edited in the hippocampus during sepsis were significantly enriched in abnormal behaviours related to social withdrawal, aggression, investigation, learning and fear/anxiety, with a seven-gene cluster of interconnected networks and mainly contributing to such functions (Fig. 2F). A missense site p.M620V in *Grik2* and a 3′UTR site in *Gria2* showed differential editing during sepsis. *Grik2* (also called *GluR6*) and *Gria2* (also called *GluR2*) belong to glutamate receptors, the predominant excitatory neurotransmitter receptors in the mammalian brain, and are activated in various neurophysiologic processes. *Grik2* and *Gria2* mRNAs are subjected to A-to-I RNA editing in multiple sites, an important mechanism that increases the functional diversity of the receptor.^42^*Camk2a* encodes an enzyme that belongs to the serine/threonine protein kinase family and regulates the plasticity at glutamatergic synapses. The alpha chain of *Camk2a* is required for hippocampal long-term potentiation and spatial learning. Defects of *Camk2a* function could cause neurodevelopmental disorders.^43^ Pathogenic variants in *FLNA*, which encodes filamin alpha essential for cell locomotion, have been linked to neurological disorders, such as bilateral periventricular nodules and heterotopias.^44^ FMR1 protein (FMRP) depletion resulted in elevated BMPR2 activity, which was reported in the prefrontal cortex of patients with fragile X syndrome.^45^ Truncating variants in the *SHANK1* gene were linked with various neurodevelopmental disorders.^46^ Additionally, studies on *Shank1*^−/−^ mice exhibited high-level anxiety and deficits in social cognition related to autism spectrum disorder (ASD).^47^ Mutations in *MAP3K7* have been reported in ASD and related neurodevelopmental disorders. In addition to these seven genes, other genes involved in cognitive function and neurodevelopment disorders, especially ASD-related features, were also differentially edited during sepsis (Supplementary Table 4). For example, *CIC* was reported as an ASD candidate gene, and its defects were linked to abnormal social behaviour and neurodevelopment symptoms. *TCF4* was associated with schizophrenia, intellectual disability and autism. A genome-wide association study in 1.1 million individuals reported that *CPNE7* variants were associated with cognitive performance measurement.^48^

Our results also identified a site in neurofibromin 2 (*Nf2*), which showed significant DRE in the hippocampus in both CLP-induced and LPS-induced sepsis. The role of *Nf2* in sepsis remains unclear. The gene encodes a probable regulator of the Hippo/SWH (Sav/Wts/Hpo) signalling pathway and could play a pivotal role in tumour suppression by restricting proliferation and promoting apoptosis.^49^ Mutations in this gene are associated with neurofibromatosis type II, characterized by nervous system and skin tumours and ocular abnormalities.^50^ The exact role of *Nf2* and its RNA editing remains to be further determined.

Consistent with humans, RNA editing sites are mainly located in short interspersed elements (SINEs), which are highly conserved in the genomes of eukaryotes.^51^ The major subfamilies of SINEs in mice include B1, B2 and B4. The repeat most overlapped with our overall RNA editing and DRE sites belonged to the B1 family, which originates from the 7SLRNA component of the common ancestor of primates and rodents.^52^ Such findings showed that RNA editing is more likely to occur on B1 repetitive elements in mice, consistent with previous reports in humans that SINE RNA may form dsRNA structures and enable ADAR enzymes to bind to and edit RNA efficiently.^53^

In addition to protein-coding genes, long intergenic non-coding RNAs (lincRNAs) have been found to influence the process of sepsis and SAE significantly.^54^ Our results showed that three lincRNA genes were also differentially edited in the hippocampus during sepsis, including *Miat*, *Snhg11* and *Meg3* (Supplementary Table 1). Miat, first discovered as a risk factor for myocardial infarction,^55^ was upregulated in septic rats,^56^ could promote inflammation and oxidative damage and may serve as a potential biomarker and therapeutic target for sepsis-induced myocardial dysfunction.^57^ By far, the role of *Meg3* in sepsis remains controversial. Studies have shown that *Meg3* can relieve intestinal injury caused by LPS-induced sepsis by regulating miR-129-5p and surfactant protein D.^58^ However, *Meg3* was also reported as a biomarker for predicting increased systemic inflammation, risk, disease severity and poor prognosis of sepsis.^59,60^ Importantly, our previous study suggested that altered *Meg3* and *Snhg11* RNA editing in the dorsal striatum could be associated with repeated winning and losing experiences in chronic social conflicts.^41^ In the current study, both genes were differentially edited during sepsis. Such findings thus indicated the potential role of dynamic RNA editing in these lincRNAs during sepsis and SAE.

Although the CLP sepsis model is more complex and possibly more physiologically relevant, LPS injection can achieve similar haematologic alterations and mortality rates. In line with this, a subset of DRE sites was shared between them, supporting the role of such consensus DRE in the hippocampus associated with the disease. Moreover, several genes differentially edited in sepsis mouse models were previously found to be differentially edited in the post-mortem brain of septic patients, including HAUS augmin-like complex subunit 2 (*Haus2*), ftx transcript (*Ftx*), protein phosphatase 1K (PP2C domain containing) (*Ppm1k*), kinesin light chain 2 (*Klc2*) and ring finger protein 168 (*Rnf168*). Such findings thus pointed to a conserved role RNA editing played during mammals’ sepsis.

Age and sex have been reported to influence the risk of sepsis and SAE. The incidence of sepsis is dramatically increased in elderly adults, especially those over 65 years old, and the age of septic patients is an independent predictor of mortality.^9^ Additionally, in elderly adults, men could be at an increased risk of sepsis and mortality.^61^ Our findings showed supporting evidence for divergent changes in A-to-I RNA editing related to age and sex during sepsis and added to the understanding of the observed clinical differences in sepsis development and outcomes.

Fullerenol has been shown to have various biological effects, such as antioxidant, anti-inflammatory and neuroprotective activities. Although fullerenol is not currently considered the gold standard antidepressant commonly used in sepsis, it was recently reported to show antidepressant-like characteristics in an LPS-induced sepsis and depression mouse model.^17^ Fullerenol pre-treatment was reported to prevent a reduction in sucrose preference. Such antidepressant-like characteristics could be due to its effects of downregulating neuroinflammation. By analysing the hippocampal RNA-seq data set of the study, our results indicated that fullerenol pre-treatment showed effects on sepsis-associated RNA editing by partially rescuing sepsis-associated RNA editing alterations, such as the p.M620V missense editing in *Grik2*, a gene under-edited in bipolar disorder.^62^ Such findings implicate the potential therapeutic potential of RNA editing in SAE and warrant further investigation of the effects of more antidepressants on sepsis-associated RNA editing in future studies.

There are a few limitations in our current study. First, the original studies of the RNA-seq included in our study had a small sample size. Larger samples with more parameters considered in future studies could help identify more factors affecting RNA editing in SAE. Second, while changes in RNA editing in the hippocampus were observed on Days 1 and 4 after sepsis induction using the CLP model, RNA editing changes over a longer period remain to be explored to further understand the effect of sepsis duration. Third, selective serotonin reuptake inhibitors like fluoxetine, sertraline and paroxetine are widely used and studied antidepressants. Exploring how selective serotonin reuptake inhibitors impact RNA editing in SAE, in comparison to fullerenol to evaluate the effects of different antidepressants on SAE-related RNA editing, could be valuable and requires further investigation.

In conclusion, our investigation in the hippocampus of sepsis mouse models demonstrates evident and dynamic alterations and influencing factors in A-to-I RNA editing and possible effects of age and sex on it. Such findings, together with our previous report on septic patients, underscore the pivotal role of RNA editing in sepsis and SAE.

## Supplementary Material

fcae260_Supplementary_Data

## Data Availability

The article presents original contributions that are included in the study. For any additional inquiries or questions, they can be directed to the corresponding author of the study [additional information (URLs): Gene Expression Omnibus (GEO) database (https://www.ncbi.nlm.nih.gov/geo/); Ensembl Variant Effect Predictor (VEP) (https://www.ensembl.org/vep); REDIportal V2.0 database (http://srv00.recas.ba.infn.it/atlas/index.html); and STRING database (http://string-db.org)].
