# Supplementary material for: Hippocampal adenosine-to-inosine RNA editing in sepsis: dynamic changes and influencing factors
Source: Brain Commun. 2024 Aug 8;6(4):fcae260. doi: 10.1093/braincomms/fcae260 (PMC11317967; doi:10.1093/braincomms/fcae260)
Supplement: fcae260_Supplementary_Data [file fcae260_supplementary_data.zip › Supplementary_Figures_1-3.pdf]

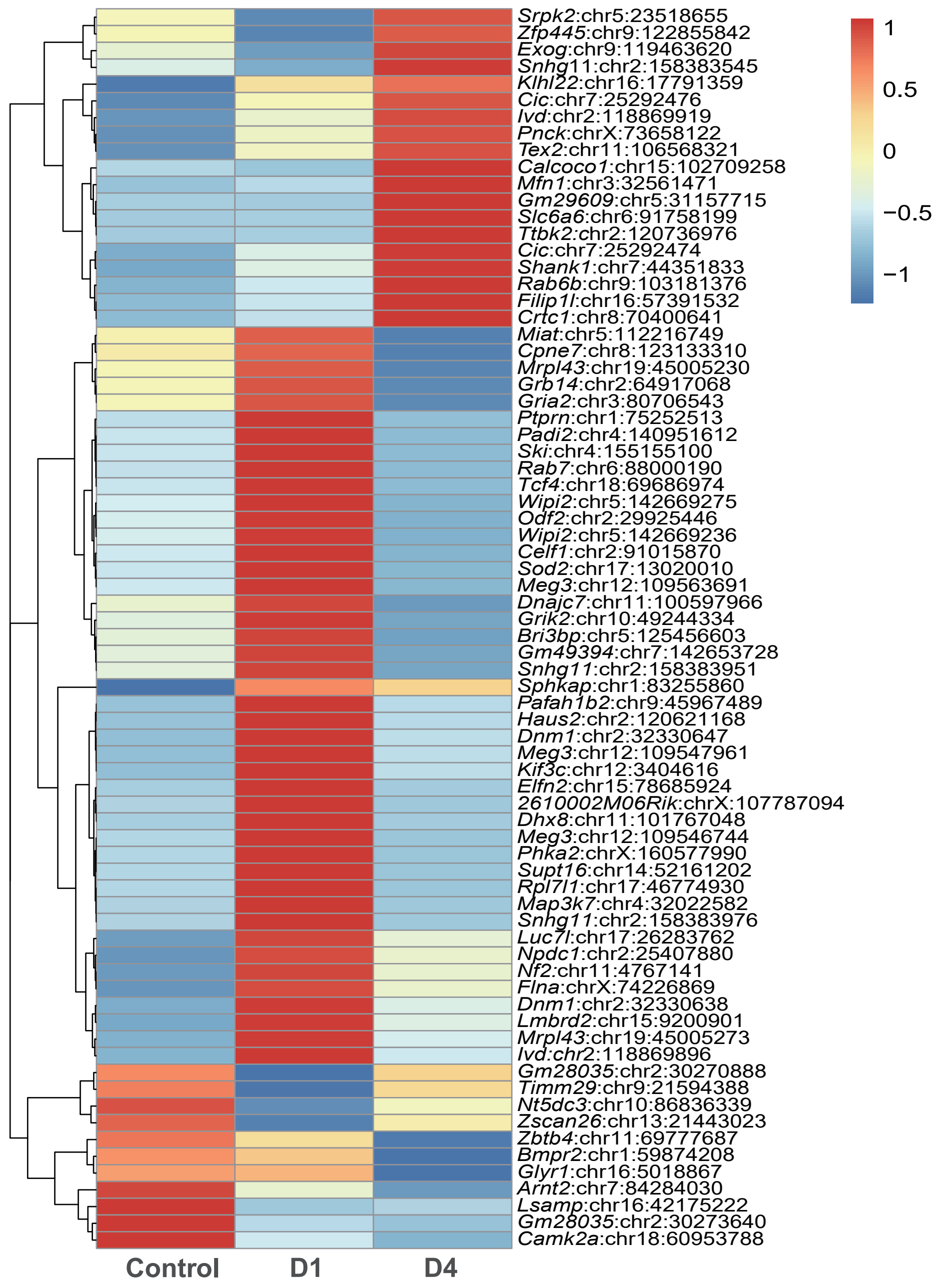

Supplementary Figure 1. Heat maps of 74 DRE sites among the three groups. *P*-values are calculated using the GLM model and LRT.

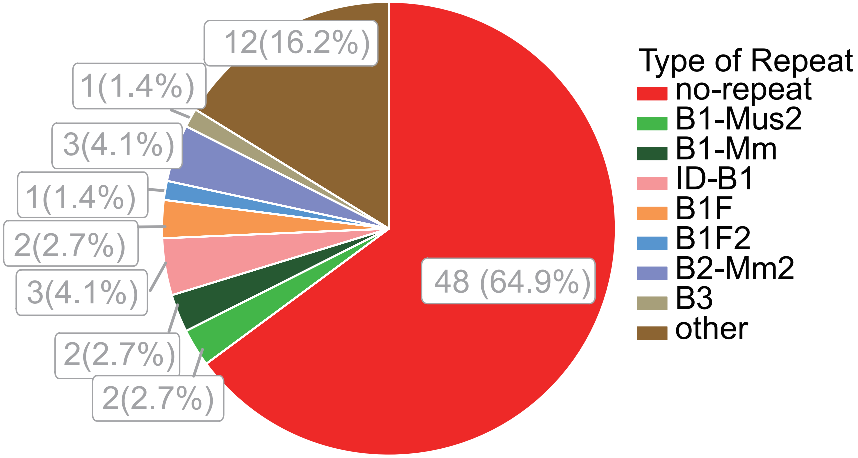

Supplementary Figure 2. Statistics of differential A-to-I RNA editing variants' overlapping repeats.

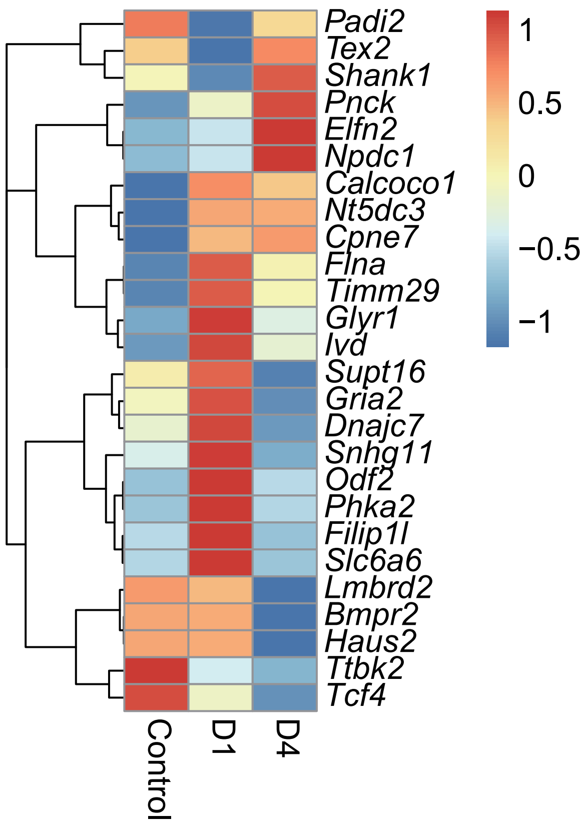

Supplementary Figure 3. **Heat maps of 26 genes shared between differentially edited and differentially expressed genes.**
